# Supplementary material for: Offloading under cognitive load: Humans are willing to offload parts of an attentionally demanding task to an algorithm
Source: PLoS One. 2023 May 19;18(5):e0286102. doi: 10.1371/journal.pone.0286102 (PMC10198496; doi:10.1371/journal.pone.0286102)
Supplement: S1 File — (PDF) [file pone.0286102.s001.pdf]

Prior instruction for Experiment 2 & Questionnaires

Prior instruction for the Joint condition in Experiment 2

Original wording in German (see manuscript for English version):

Bevor wir mit den Trainingsdurchläufen beginnen, zunächst noch eine wichtige Information vorab: Bei dem sogenannten Computerpartner handelt es sich um eine Software, die speziell für diese Aufgabe entwickelt wurde. Der Computerpartner wurde so programmiert, dass er die Aufgabe mit 100%-iger Korrektheit löst, egal wie viele Punkte er verfolgen muss.

Below, we present the set of questionnaire items that was used in both experiments. We display the original wording in German and provide links to the English versions for the Affinity for Technological Systems, Trust in Automation, and Desire to Control questionnaires.

Task-related questions

Im Folgenden stellen wir Ihnen zunächst einige Fragen zu Ihren Erfahrungen mit der Aufgabe im Experiment. Sie können Ihre Antworten hier frei formulieren.

1. Als Sie die Aufgabe alleine bearbeitet haben:
   a. Wie viele Kreise haben Sie verfolgt, und warum haben Sie diese Anzahl gewählt?
   b. Welche Kreise haben Sie verfolgt, d.h. haben Sie bei der Auswahl der Kreise eine bestimmte Strategie verfolgt?
2. Als Sie die Aufgabe gemeinsam mit dem Computerpartner bearbeitet haben:
   a. Wie viele Kreise haben Sie verfolgt, und warum haben Sie diese Anzahl gewählt?
   b. Welche Kreise haben Sie verfolgt, d.h. haben Sie bei der Auswahl der Kreise eine bestimmte Strategie verfolgt?
   c. Was waren Ihre Gedanken hinsichtlich der Aufgabenteilung mit dem Computerpartner? Wie haben Sie entschieden, wie viele Kreise Sie selbst verfolgen und wie viele Sie dem Computerpartner überlassen?

3. Wie schätzen Sie die Kapazität des Computerpartners ein? Konkret: Wie viele Kreise kann der Computerpartner akkurat verfolgen - was denken Sie?
4. Haben Sie noch weitere Kommentare oder Anmerkungen zum Experiment? Teilen Sie uns diese hier gern mit.

Affinity for Technological Systems (Franke, Attig, & Wessel, 2019)

The English version can be found in Franke, Attig, & Wessel (2019).

Im Folgenden geht es um Ihre Interaktion mit technischen Systemen. Mit "technischen Systemen" sind sowohl Apps und andere Software-Anwendungen als auch komplette digitale Geräte (z.B. Handy, Computer, Fernseher, Auto-Navigation) gemeint. Bitte geben Sie den Grad Ihrer Zustimmung (von "stimmt gar nicht" bis "stimmt völlig") zu folgenden Aussagen an.

(Antwortoptionen: Stimmt gar nicht; Stimmt weitgehend nicht; Stimmt eher nicht; Stimmt eher; Stimmt weitgehend; Stimmt völlig)

1. Ich beschäftige mich gern genauer mit technischen Systemen.
2. Ich probiere gern die Funktionen neuer technischer Systeme aus.
3. In erster Linie beschäftige ich mich mit technischen Systemen, weil ich muss.
4. Wenn ich ein neues technisches System vor mir habe, probiere ich es intensiv aus.
5. Ich verbringe sehr gern Zeit mit dem Kennenlernen eines neuen technischen Systems.
6. Es genügt mir, dass ein technisches System funktioniert, mir ist es egal, wie oder warum.
7. Ich versuche zu verstehen, wie ein technisches System genau funktioniert.
8. Es genügt mir, die Grundfunktionen eines technischen Systems zu kennen.
9. Ich versuche, die Möglichkeiten eines technischen Systems vollständig auszunutzen.

Trust in Automation (Körber, 2019)

The English version can be found here:
https://github.com/moritzkoerber/TiA_Trust_in_Automation_Questionnaire/blob/master/Trust-in-Automation_TiA_questionnaire.pdf

Note that the following three items are only a subset of the full set of items of this questionnaire.

Im Folgenden geht es um Ihr generelles Vertrauen in technische Systeme. Bitte geben Sie den Grad Ihrer Zustimmung (von "stimme gar nicht zu" bis "stimme voll zu") zu folgenden Aussagen an.

(Antwortoptionen: Stimme gar nicht zu; Stimme eher nicht zu; Stimme weder zu noch nicht zu; Stimme eher zu; Stimme voll zu)

1. Bei unbekannten technischen Systemen sollte man eher vorsichtig sein.
2. Ich vertraue einem technischen System eher, als dass ich ihm misstraue.
3. Technische Systeme funktionieren generell gut.

Reliability and competence of computer partner

Items were adapted from the Trust in Automation questionnaire (Körber, 2019).

Im Folgenden geht es um Ihre Einschätzung des Computerpartners. Mit "Computerpartner" ist hier das Computerprogramm gemeint, mit dem Sie gemeinsam die Aufgabe im 2. Teil des Experiments bearbeitet haben. Bitte geben Sie den Grad Ihrer Zustimmung (von "stimme gar nicht zu" bis "stimme voll zu") zu folgenden Aussagen an

(Antwortoptionen: Stimme gar nicht zu; Stimme eher nicht zu; Stimme weder zu noch nicht zu; Stimme eher zu; Stimme voll zu)

1. Der Computerpartner arbeitet zuverlässig.
2. Ich vertraue dem Computerpartner.
3. Ein Ausfall des Computerpartners ist wahrscheinlich.
4. Der Computerpartner kann wirklich komplizierte Aufgaben übernehmen.
5. Ich kann mich auf den Computerpartner verlassen.

6. Der Computerpartner könnte stellenweise einen Fehler machen.
7. Ich bin überzeugt von den Fähigkeiten des Computerpartners.

Desirability of Control Scale (Burger & Cooper, 1979)

The English version can be found in Burger & Cooper (1979).

Im Folgenden geht es um Ihre Selbsteinschätzung. Bitte geben Sie den Grad Ihrer Zustimmung (von "trifft überhaupt nicht zu" bis "trifft immer zu") zu folgenden Aussagen an.

(Antwortoptionen: Trifft überhaupt nicht zu, Trifft normalerweise nicht zu, Trifft meistens nicht zu, Trifft mal zu, mal nicht zu, Trifft meistens zu, Trifft normalerweise zu, Trifft immer zu)

1. Ich bevorzuge eine Arbeit, bei der ich viel Kontrolle habe über das, was ich tue und wann ich es tue.
2. Ich beteilige mich gern politisch, weil ich so viel Mitspracherecht wie möglich haben möchte.
3. Ich versuche, Situationen zu vermeiden, in denen jemand anderes sagt, was ich tun soll.
4. Ich möchte lieber Anführer als Mitläufer sein.
5. Ich genieße es, die Handlungen anderer beeinflussen zu können.
6. Ich überprüfe sorgfältig alles an einem Auto, bevor ich eine lange Reise antrete.
7. Andere wissen in der Regel, was das Beste für mich ist.
8. Ich treffe gerne meine eigenen Entscheidungen.
9. Ich nehme mein Schicksal gern selbst in die Hand.
10. Ich würde es vorziehen, wenn jemand anderes die Führungsrolle übernimmt, wenn ich an einem Gruppenprojekt beteiligt bin.
11. Ich halte mich im Allgemeinen für fähiger als andere Personen im Umgang mit schwierigen Situationen.
12. Ich führe lieber mein eigenes Geschäft und mache meine eigenen Fehler, als auf die Anweisungen anderer Personen zu hören.

13. Ich möchte eine gute Vorstellung davon bekommen, worum es bei einer Arbeit geht bevor ich anfange.
14. Wenn ich ein Problem sehe, tue ich lieber etwas dagegen anstatt tatenlos zuzusehen, wie das Problem weiterbesteht.
15. Ich würde lieber Anweisungen geben als sie zu erhalten.
16. Ich wünschte, ich könnte viele der täglichen Entscheidungen des Lebens auf jemand anderen abwälzen.
17. Wenn ich Auto fahre, versuche ich, mich nicht in Situationen zu bringen, in denen ich von einer anderen Person verletzt werden könnte.
18. Ich ziehe es vor, Situationen zu vermeiden, in denen jemand mir sagen muss, was ich zu tun habe.
19. Es gibt viele Situationen, in denen ich lieber nur eine einzige Möglichkeit hätte, als eine Entscheidung zwischen mehreren Möglichkeiten treffen zu müssen.
20. Ich warte gerne ab, ob jemand anderes ein Problem lösen wird, damit ich mich nicht selbst darum kümmern muss.
