# Supplementary material for: Offloading under cognitive load: Humans are willing to offload parts of an attentionally demanding task to an algorithm
Source: PLoS One. 2023 May 19;18(5):e0286102. doi: 10.1371/journal.pone.0286102 (PMC10198496; doi:10.1371/journal.pone.0286102)
Supplement: Attachment — Submitted filename: ResponseToReviewers_R1_PLOSONE_SUBMIT.docx [file pone.0286102.s002.docx]

**Revision for manuscript PONE-D-23-00394**

Algorithmic appreciation under cognitive load: Humans are willing to offload parts of an attentionally demanding task to an algorithm

**Editor Comments:**

*Both reviewers agree that the manuscript will provide a valuable contribution to the discourse when revised. When revising the manuscript make sure to answer to all comments and include even the minor points. I would like to draw particular attention to the following points:*

**Response:**

We’d like to thank you and the two reviewers for evaluating our work! Please find our replies to all comments below. **Please note that references to page numbers refer to the marked-up version of the manuscript.**

*Interpretation of results:*

*Reviewers 1 and 2 comment on several issues regarding the interpretation of results. With the task on hand being of relatively low valence, does the experiment really measure algorithmic aversion? Without comparison to a human partner, does the experiment really measure algorithmic aversion? In your revision, please make sure to reflect on these issues and draw on additional examples from the literature regarding low valence tasks. Also focus on cognitive load vs. maximum cognitive capacity when comparing results from experiments 1 and 2.*

**Response:**

We thank the reviewers for raising these issues. We completely agree that considering the valence of the task (i.e., the difference between low vs. high stakes scenarios) is important and we now acknowledge this at several points throughout the manuscript (pages 5-6, 24). We have also added relevant literature that was missing from the original version of the manuscript (pages 3-4). Moreover, we acknowledge that not having a human comparison is a limitation of our study, allowing us to only draw limited conclusions regarding algorithmic aversion. We discuss this in depth in the Discussion (page 24). Note that we also use the term algorithmic appreciation / aversion with more care (e.g., we changed the title and abstract and removed parts of the discussion (page 23). Finally, we revised our use of the terms cognitive load vs. cognitive capacity. We describe the observed effects only in terms of a reduction of cognitive effort/load.

*Trustworthiness of results:*

*Please also make sure to reflect on comparisons between experiments 1 and 2 as there are some concerns that they measure something completely separate. Be wary of the type 1 errors - with sometimes barely significant results (p = .044) and different experimental settings your study is not replicated yet. Without replicating results this might have been a random finding. Please double-check to not introduce false positive findings into the literature.*

**Response:**

We agree that the reported results you point to here were not particularly strong, statistically speaking, and we cannot be sure whether they would be replicated. We have therefore decided to remove the analysis (and the corresponding interpretation) altogether. We have also addressed the concern that the experiments measure something separate in the Discussion (page 22).

*Accuracy of reporting:*

*Both reviewers find several instances where signs seem to be opposite than expected or you describe majorities when minorities were more suitable. Please double-check the results and interpretation section carefully and ensure to accurately describe all results (also regarding description of references).*

**Response:**

We thank the reviewer for spotting these mistakes. We have corrected all of them.

*Open data:*

*Please provide the dataset and analysis syntax open without restrictions to comply with PLOS ONE publication criteria.*

**Response:**

Our full data set and the analysis script are now available on OSF: https://osf.io/qbgm3/?view_only=d5cd63e768d149e48d99e68292ea2609

**Reviewers' comments:**

**Reviewer #1**

*Based on previous work showing variability in algorithmic aversion – where despite widespread use of smartphone apps (such as Siri or Google Maps), individuals still do not trust an algorithm to make medical assessments or forecasting as much as a human, or at least in an unsupervised way. The current work tests to see whether cognitive load can influence algorithmic aversion. The Authors use a multiple object tracking (MOT) task which requires participants to choose how many targets they would like to track and then after the objects move, they are asked to select the original targets. By first having participants perform the task unassisted, they develop a baseline of participant behavior and allow the participants to evaluate their own abilities/working memory capacity. Subsequently, participants are given the opportunity to let a computer partner track some of the objects and offload the work of the task to the algorithm. In Experiment 1, participants are not told how accurate the computer partner is, but in Experiment 2, participants are explicitly told that the algorithm has a 100% success rate. By comparing the relative willingness to offload work to the computer based on accuracy (i.e., Experiments 1 vs. 2), the current studies test the hypothesis that individuals are more likely to let a computer partner do some of the work if they believe it to perform well. The Authors conclude that individuals will partially offload task demands to a computer if the task exceeds their own cognitive capacity. The evidence for this comes primarily from the “how many objects tracked” results of participants in the Solo vs. Joint conditions: where across both Experiment 1 and 2, participants chose to track fewer objects when the computer partner was able to assist relative to when they were doing so themselves. They also conclude that in certain conditions, individuals will be more likely to offload work if the computer partner is assumed to be more accurate. This effect wasn’t supported by a direct comparison of Experiment 1 vs 2 but comes from the observation that when the data were split into sets of 10-trials, the number of objects chosen in the Joint task between Exp 1 and 2 were different in the first set of 10 trials. Additionally, several personality traits were examined that might affect participants’ willingness to offload work to the computer partner, though none were significantly related to this.*

*I think the article has several key strengths. In terms of motivating the current research question, the point that the “humans tend to commit more errors under high attentional load and could thus substantially profit from cognitive offloading” is a good one and motivates the current work nicely. The figures are great - Figure 1 is quite helpful for displaying the task design. I particularly like the way the data is presented in the plots in Figures 2-7. I appreciate that the researchers show the distribution of the data by plotting the individual data points! For the most part, the study is well designed, and the Authors have considered many of the potential limitations and pro-actively addressed them. I also think that with a couple exceptions, the conclusions are reasonable and well calibrated based on the results. Though not the primary goal of the study per se, I found it interesting that the participants didn’t trust the computer more in Exp 1. I have a few concerns about alternative explanations for this finding (see Major point #3 below) but an interesting result nonetheless.*

*For me, the major weaknesses relate to how this research is framed (Major points #1 & #4 below), the conclusions drawn from a very weak effect (Major point #2 below), and the fact that the data and code are not openly available (Major point #5).
My overall assessment is that this paper does show an interesting effect and the key results are sufficiently robust, so with some modifications it will be suitable for publication. I detail these major concerns and several minor points below.*

**Response:**

We thank the reviewer for the careful review of our work. To address the reviewer’s concerns, we have (1) improved the framing of our research, (2) removed the analysis that reported only a very weak effect, and (3) have made our data and code openly available. Please find our detailed responses below.

*Major Concerns:*

*1. Intro: I am not an expert in human-computer interactions so I’m not sure if there are any missing or inappropriate references for the background. However, some of the examples presented that are evidence for algorithmic aversion seem to be higher stakes than the current study (i.e., the example of a medical decision) or are not so much an accuracy decision, but an evaluation (the art authenticity example). The closest analog I see to the current study is that of using Google Maps or potentially the forecasting. It strikes me a bit as though the Authors are comparing apples and oranges a bit by lumping this all under the idea of algorithmic aversion/trust for humans over computers, when it might have something to do with risk aversion in the case of high stakes decisions. Perhaps there are more relevant, low stakes examples out there that could be drawn upon? At a minimum, including a discussion of other contextual factors that could influence algorithmic aversion (i.e., risk aversion, affective influences on decision making, whether the human is another person or oneself), would be helpful to situate the current study.*

**Response:**

We have added a thorough discussion of other contextual factors in the Introduction, also citing additional literature (pages 3-4). In particular, we discuss the difference between low vs. high stake scenarios and point out that the results of our study cannot generalize to high stakes scenarios (pages 5-6, 24).

*2. Results: The seemingly exploratory analyses comparing Exp 1 and 2 don’t appear to control for multiple comparisons at all. I suggest including MC correction for these results and removing the discussion of computer partner accuracy as a contributing factor, as the initial null result (comparing overall Exp 1 and Exp 2) and the weak effect for the first 10 trials (p = 0.044 uncorrected) doesn’t sugest a particularly robust or reliable effect.*

**Response:**

We agree that the effect does not seem particularly reliable and have therefore removed the exploratory analysis and the corresponding interpretation of the weak effect. (Note though that the control for multiple comparisons was not necessary as all comparisons were performed within a linear mixed model and not as separate t-tests).

*3. Methods/Results: For the question of how many targets did participants think that the computer partner was able to track – I can see why the Authors did things in the order they did (i.e., question asked after all study procedures in Exp 1 and before starting joint condition in Exp 2). However, I wonder if this muddies whether conclusions can be drawn from comparing the number of items they assumed the computer could track between Experiments 1 and 2. That is, some participants in Exp 1 may have been making an absolute judgment (answering the question of “how accurate is the computer irrespective of what the participant did?”) and some might be reporting this based on what they saw: 6 minus the number that they usually chose. Maybe there is some additional evidence that I’m missing that mitigates this explanation. Or maybe there are some analyses that could be done to examine this (perhaps looking at the responses in Fig 3 as a function of number chosen)? Otherwise, I think it should be noted as a limitation.*

**Response:**

We would like to clarify that there has been a misunderstanding regarding the order in which the question about the computer’s capacity was asked. In fact, the question was always asked AFTER the joint condition; there was no difference between Experiment 1 and Experiment 2. We believe that this should resolve the reviewer’s worry about potential order effects. Still, we followed the reviewer’s suggestion and checked whether participants’ responses to the question about computer’s capacity were correlated with participants’ number of tracked targets. This correlation was not significant (r = -0.05, *p* = 0.796), suggesting that there was no systematic relation between the two variables in question.

*4. Interpretation: There is some inconsistency in how the effects are described – as related to cognitive load or as exceeding cognitive capacity. For example, in the discussion, “The present findings show that humans are willing to (partially) offload task demands to an algorithm if these demands exceed their own attentional capacity, providing evidence of algorithmic appreciation (rather than aversion).” It seems to me that the data strongly support the idea that the participants did so to decrease their cognitive load, not because it exceeds their cognitive capacity (as in the Solo condition they were presumably at their capacity, and this is a decrease from that). I would recommend changing any discussion of capacity to effort/cognitive load based on this.*

**Response:**

We acknowledge this inconsistency and agree that only the description in terms of “cognitive load” is consistent with the data. Thus, we have changed the relevant passages such that we describe the observed effects only in terms of a reduction of cognitive effort/load.

*5. Data & code availability: The data are available for review but not openly available. In all my reviews, I request that the Authors upload their data and analysis code to a public repository to facilitate transparency and reproducibility of results.*

**Response:**

We have made all data and the analysis script available on OSF: https://osf.io/qbgm3/?view_only=d5cd63e768d149e48d99e68292ea2609

*Minor Concerns:*

*1. Results paragraph 1: “We found that participants chose to track significantly fewer targets in the Solo (M = 3.42, SD = 0.55) compared to the Joint (M = 2.68, SD = 1.08) condition (t(25) = -3.67, p = .001, Cohen’s d = -0.72), indicating that, in the Joint condition, participants decided to offload a subset of targets to the computer partner.” Are these condition means meant to be in the opposite order?*

**Response:**

We thank the reviewer for pointing this out and have corrected the mistake.

*2. The experimental setup is described well and clearly. I think a bit more detail is needed on the analyses. It would be helpful to clearly state what the between and the within subjects effects were in any regressions. It would also help to know what software was used for the statistical analyses. I’m confused by the negative Cohen’s d values so I’m wondering if this is an oddity of a particular analysis software (or some R or python package/ library). These should be changed to positive values in any case.*

**Response:**

We now explicitly state the between and within subjects effects in the regression analyses; we report which software packages were used, and we have changed the Cohen’s d values so that all of them are positive.

*3. It would be useful if the authors described the magnitude of the effects as well as I assume most folks don’t have the Cohen’s d scale memorized.*

**Response:**

We have added the magnitudes of the effects in all cases.

4. “When comparing the frequency of the reported strategies in the Joint condition across *experiments (for a descriptive overview, see Figure 7), we observed that the majority (i.e., 11 out of 26 participants) indicated no change in behavior relative to the Solo condition in Experiment 1 whereas in Experiment 2, the majority (i.e., 11 out of 26 participants) reported minor offloading.” 11 out of 26 isn’t a majority of participants, do the Authors mean this was the most common response?*

**Response:**

These proportions were indeed described incorrectly; we have changed the wording accordingly (page 18).

*5. On page 17, I realize these are null results, but it would still be useful for the researchers to report beta values for the multiple regression in addition to or instead of the t-stats.*

**Response:**

We have added beta values for the multiple regression analysis.

*Other points: As I do in all my reviews, I ran statcheck on this manuscript and found no inconsistencies.*

**Response:**

We thank the reviewer for checking for inconsistencies.

**Reviewer #2**

*Thank you for the opportunity to review this manuscript. It is an interesting read and a well-composed study with convincing analyses. There are some issues that raised my concern which may well be solved quickly.*

*If I understand correctly, in Joint, participants had another implicit task in Exp1 namely evaluating the performance of the partner to be able to decide how many objects to offload and thus to maximize the “points” they achieve together with the partner (this was set to 100% in Exp2 so no second task here). If in Exp1 Solo they chose as many objects as humanly possible (“in the Solo condition, participants tracked between 3 and 4 targets on average, indicating that they tracked (close to) the maximum number humans are typically capable of tracking [15]”), they would then not be able to keep up this high number of objects in Joint but they would have to reduce this number to achieve their additional second task, evaluating the partner. If this is the case, then Exp1 and Exp2 would be hard to compare. Also, considering this second implied task, participants did not really offload any objects in Exp1 Joint but in addition to their own objects also had to evaluate ALL other objects because they were selected automatically to be tracked by the partner. If this interpretation of mine is incorrect it needs to be clarified in the text so the reader does not make the same mistake.*

**Response:**

We fully understand the reviewer’s concern and address it at length in the Discussion (page 22). Briefly, from participants’ questionnaire responses of Experiment 1, we know that indeed several (i.e., 19%) participants tried to find out about the computer’s accuracy through monitoring the targets selected by the computer, which confirms your suspicion. However, participants quickly realized that the computer’s accuracy was high and stopped monitoring after only a few trials (no one monitored the computer partner throughout the experiment). These subjective self-reports are supported by participants’ objective accuracy scores: if participants had monitored the computer throughout the entire experiment, then their own performance should have been severely impaired. Instead, participants’ performed with the same average accuracy of 97% in Experiment 1 and 2, while the number of tracked targets was similar. The fact that accuracy levels did not differ between experiments suggests that participants did not perform a secondary task in Experiment 1.

*I did not find any information on the actual accuracy of the partner (algorithm) in Exp1 but may have overlooked it. Assuming its accuracy is perfect, does not accuracy need to be higher in Joint because the partner automatically takes care of the objects not selected by the participant?*

**Response:**

We report accuracy only for the participants’ performance (in both the Individual and the Joint condition) so that it is comparable across conditions. We have clarified this in the caption of Figure 2. The accuracy of the computer is 100% in both experiments; we have clarified this on page 11.

*With regard to the general design of the study, is it really possible to conclude anything about algorithm appreciation or aversion? There is just this one type of partner (i.e., the algorithm) and no comparison against the same design with a human partner. It may well be that the participants would have behaved exactly the same when partnered with another human (in Exp1 evaluating their performance, in Exp2 trusting the claimed accuracy). This was my impression of some of the literature summarized in the introduction as well.*

**Response:**

We acknowledge this limitation of our study and discuss it in depth in the Discussion (page 23). Note that we also use the term algorithmic appreciation / aversion with more care (e.g., we changed the title and abstract and removed parts of the Discussion (page 23)).

*Twice: “majority (i.e., 11 out of 26 participants)” needs to be minority?*

**Response:**

Thanks for spotting this error; we have corrected it.

*Conference contributions better described as such instead of “Research Papers. 168.”*

**Response:**

We have changed the reference accordingly.

*In “We found that participants chose to track significantly fewer targets in the Solo (M = 3.42, SD = 0.55) compared to the Joint (M = 2.68, SD = 1.08) condition” here fewer needs to be more?*

**Response:**

Thanks again for spotting this error; we have corrected it.

*It’s a well-composed study and convincing to read with well-performed analyses, so I hope my questions can be answered quickly.*

**Response:**

Thanks so much; we hope we could provide satisfactory responses to all questions raised.
